# Improving Nutrition Information in the Eastern Mediterranean Region: Implementation of Front-of-Pack Nutrition Labelling

**DOI:** 10.3390/nu12020330

**Published:** 2020-01-26

**Authors:** Ayoub Al-Jawaldeh, Mike Rayner, Chantal Julia, Ibrahim Elmadfa, Asmus Hammerich, Karen McColl

**Affiliations:** 1 WHO Regional Office for Eastern Mediterranean Region, Cairo 11371, Egypt; 2 Centre on Population Approaches for Non-Communicable Disease Prevention, Nuffield Department of Population Health, University of Oxford, Oxford OX1 2JD, UK; 3 Nutritional Epidemiology Team, UMR 1153 Inserm, U1125 INRA, CNAM Paris 13 University, Center of Research in Epidemiology and Statistics (CRESS), Bobigny, 75003 Paris, France; 4 Department of Nutritional Sciences, Faculty of Life Sciences, University of Vienna, 1010 Wien, Austria; 5 Freelance Writer and Policy Consultant, 21 Apple Grove, Bognor Regis, West Sussex PO21 4NB, UK

**Keywords:** nutrition, nutrition labelling, front-of-pack, FOP, healthy diet, Eastern Mediterranean Region

## Abstract

The provision of simplified nutrition information, in a prominent place on the front of food packages, is recommended as an important element of comprehensive strategies to tackle the burden of death and disease caused by unhealthy diets. There is growing evidence that front-of-pack nutrition labels are preferred by consumers, are more likely to be looked at or noticed than nutrition labelling on the back or side of packages and can help consumers to better identify healthier and less healthy products. This review summarizes current implementation of front-of-pack nutrition labelling policies in the countries of the WHO Eastern Mediterranean Region. Implementation of front-of-pack nutrition labelling in the Eastern Mediterranean Region remains limited, but three types of scheme were identified as having been implemented or at an advanced stage of development by governments in six countries. Through a review of reviews of existing research and evidence from country implementation, the authors suggest some pointers for implementation for other countries in the Region deciding to implement front-of-pack nutrition labelling policies.

## 1. Introduction

In the WHO Eastern Mediterranean Region half the Region’s adult women (50.1%) and more than two in five men (43.8%) are overweight or obese and more than 2.2 million lives are lost each year to noncommunicable diseases (NCDs) [1]. Unhealthy diets are a major contributor to this burden, estimated to be responsible for over 20% of adult deaths in the Region [2]. All available policy tools should be leveraged to improve diets, and policies to improve access to nutrition information—including on food labels—are an important element of such efforts.

The provision of nutrition labelling on prepackaged foods has long been recognized as being one means of empowering consumers to make healthier food choices. WHO has recommended countries use food labelling to tackle malnutrition in all its forms and to help meet the global targets on nutrition and NCDs [3,4,5,6]. In the Eastern Mediterranean Region, the Regional Framework for Obesity Prevention, adopted by WHO Member States in 2018, recommends implementation of front-of-pack nutrition labelling for all prepackaged foods [7].

To date, nutrition information has largely been implemented as a detailed nutrient content declaration (sometimes known as a nutrition information panel), which declares the amount of key nutrients in the food, usually in small print size, on the back or side of food packages. The international food standards body, the Codex Alimentarius Commission, recommends that Member States make labelling with nutrient declarations mandatory [8] and it is estimated that more than 50 countries have now legislated to make such labelling mandatory [9].

Such information is particularly important given the major role of highly processed, packaged foods in modern diets in the Region [10,11] and the greater difficulty that may pose for consumers to determine how much saturated or trans fats, sugars or salt such foods contain. Most of the countries in the Eastern Mediterranean Region have seen a transition from traditional diets to more “Westernised” diets and higher intakes of highly processed and fast foods [12].

There is consistent research to suggest an association between use of nutrition labels, whether on the front or back of food packages, and healthier diet [13,14,15], but actual use of labels is relatively low and certain groups—particularly women, people on higher incomes, and/or with more education, as well as those who already have a specific interest in diet and health—are more likely to use nutrition labels [13,14,16]. Widespread problems with understanding and interpretation of the relatively complex numerical information on back-of-pack nutrition information panels are also reported [13,16,17,18], particularly among people with lower socioeconomic status [13,16]. There are concerns, therefore, that reliance on back-of-pack nutrient declarations alone could widen socioeconomic inequalities in health.

Hence, there has been growing interest in the provision of supplementary, simplified nutrition information, in a prominent place on the front of package to help consumers understand the nutritional quality of foods. Such front-of-pack nutrition labelling is intended to supplement, rather than replace, more detailed nutrition information on the back or side of packs.

Globally, it is estimated that, by 2017, 1.5 billion people were living in countries where front-of-pack nutrition labelling schemes have been implemented or officially proposed [19]. Many different front-of-pack labelling schemes have emerged, with no one scheme regarded as being as optimal. To help make sense of this complex picture, some conceptual frameworks have been developed to categorize the different types of schemes [20,21,22].

This paper summarizes the current status of implementation of front-of-pack nutrition labelling in the WHO Eastern Mediterranean Region and explores how lessons from international experience could guide further implementation in the Region.

## 2. Methods

In order to build a picture of the state of implementation of front-of-pack nutrition labelling in the WHO Eastern Mediterranean Region and to inform its future implementation, a literature review was conducted for development of a background document for discussion at a WHO Technical Consultation held in Beirut, Lebanon, between 11 and 13 September 2018.

This paper builds on four elements:

- An initial scoping literature review which identified, through PubMed, review articles on nutrition labelling which were published in English after 2000 [13,14,15,16,17,18,20,22,23,24,25,26,27,28,29,30,31,32,33]. Only reviews which included any explicit consideration of front-of-pack nutrition labelling were included. In addition, relevant grey literature—including reports from official bodies such as WHO, the Codex Alimentarius Commission and the European Commission—was identified [19,20,34,35]. Further specific references were identified from these reviews to enable more in-depth exploration of some of the issues highlighted in the discussion section.
- Presentations and discussions during the Technical Consultation in September 2018.
- Information on implementation in the Eastern Mediterranean Region, identified through follow-up communication with experts about ongoing research and with nutrition or food regulatory officials in the relevant countries.
- Inclusion of new reviews published since the original literature review and technical consultation in 2018 [36,37,38].

The term “front-of-pack labelling” refers to supplementary information which is designed to assist in interpreting the information in nutrient declarations on the back of packages.

WHO defines front-of-pack labelling as referring to nutrition labelling systems that:

- Are presented on the front of food packages (in the principle field of vision) and can be applied across the packaged retail food supply;
- Comprise an underpinning nutrient profile model that considers the overall nutrition quality of the product and/or the nutrients of concern for NCD;
- Present simple, often graphic information on the nutrient content and/or nutritional quality of products to complement the more detailed nutrient declarations usually provided on the back of food packages [20].

This paper deals with nutrition labelling on prepackaged foods. There is clearly, however, also scope for provision of simplified nutrition information on retail shelves or on unpackaged foods, as well as on menus or at point-of-purchase in restaurants and other food service outlets.

Front-of-pack nutrition labelling generally has two specific objectives:

- To provide consumers with nutrition information in a more understandable format, with a view enabling them to make healthier food choices;
- To encourage food manufacturers to develop new products and reformulate their existing products towards healthier food products.

Some additional objectives are sometimes also considered:

- To improve consumer understanding about the links between the nutrient content of foods and health, particularly for the prevention of NCDs;
- To facilitate health professional advice on nutrition and healthy eating;
- To reduce consumer confusion and deception about food products, particularly in relation to misleading use of health and nutrition claims.

In relation to these objectives, the most relevant outcomes are:

- Effects on consumer understanding of the nutritional quality of foods;
- Effects on consumer purchasing behaviour;
- Changes in the nutritional composition of foods (fat, saturated fat, sugars, and salt levels).

## 3. Results

The initial literature review summarized the evidence relating to effectiveness of front-of-pack labelling, identified the different systems in place and provided an outline of front-of-pack nutrition labelling implementation globally.

The Technical Consultation and other follow-up provided a picture of implementation in the Eastern Mediterranean Region and identified some emerging messages from the evidence base to inform introduction of front-of-pack labelling in the Region.

### 3.1. Evidence for Effectiveness of Front-of-Pack Labelling

Despite a large body of research on use and interpretation of nutrition labels, including front-of-pack labelling, the reviews highlight that interpretation of findings is difficult because the studies are very heterogeneous and have been conducted on different labelling systems. While some studies compare one type of front-of-pack label with no front-of-pack label, others involve comparisons of various different types of front-of-pack label. In addition, effectiveness can be considered in terms of impact on a wide variety of outcomes including consumer attitudes towards different labelling schemes (preference, subjective assessment of consumer understanding), objective consumer understanding of labels and impact on food choices in laboratory or real-world settings. While some of these outcomes, such as consumer preference over other label formats, may be useful in the development phase for front-of-pack labelling systems, they are less relevant once the scheme has been implemented, when outcomes relating to consumer understanding, purchasing, and food product reformulation are more important for measuring impact.

A number of reviews have been conducted to summarize and interpret the evidence [13,14,15,16,17,18,23,24,25,26,27,28,34]. These reviews, like the studies themselves, differ considerably in the types of labelling examined. In addition, the implementation of front-of-pack nutrition labelling is evolving so rapidly that not much of the research incorporates the newest forms of front-of-pack labelling (e.g., Nutri-Score and warning labels) or the most up-to-date version of existing systems (e.g., UK traffic light labelling, standardized in 2013). The reviews highlight that most of the research reviewed is based on online or laboratory settings and there have been few studies in real world settings. A Cochrane review, for example, analyzed randomized or quasi-randomized controlled trials, controlled before-and-after studies and interrupted time series studies on any type of nutrition labelling published before September 2017. Many studies relating to menu labelling were included but only one real-world study in a grocery store and one real-world study on vending machines were considered suitable for inclusion, and the authors did not include the results of either, due to difficulties in interpreting the results [26]. Additionally, since the acceleration of government-led implementation of front-of-pack labelling is relatively recent, it is often too soon to have collected much evidence on impact from country experience, although such evidence is now beginning to emerge.

There is, nonetheless, a growing body of evidence showing that front-of-pack nutrition labels are preferred by consumers [13,27,34,39] and are more likely to be looked at or noticed by consumers compared to nutrient declarations/nutrition information panels on the back or side of packages [23,25,27,40]. In addition, there is increasing evidence that front-of-pack labels can help consumers better identify healthier and less healthy products, with interpretive front-of-pack labels having most consistently shown positive results [13,15,16,17,23].

Systematic reviews found mixed results of earlier studies examining impact on consumer purchases, but there is emerging evidence from more recent and comparative studies suggesting an impact on food purchases. A 2016 meta-analysis—which pooled the results of nine studies, mostly relating to front-of-pack labelling—estimated that nutrition labelling schemes would increase the number of people selecting healthier food products by around 18% [23]. In addition, increasingly, studies on recently-implemented interpretive schemes (HSR or Nutri-Score) have found that people using the labels buy foods of better nutritional quality [23,41,42,43,44], with some studies finding improvements for Nutri-Score across all socio-economic groups, with the biggest impact among those on lower incomes [42,44]. Research also suggests that results on front-of-pack labelling may improve with time, as labels become more useful to consumers as they become more familiar with them [43].

Experience from New Zealand, the Netherlands, Ecuador, and Sweden—as well as experience from the United States with mandatory trans fatty acid labelling—suggests that implementation of front-of-pack labelling can prompt manufacturers to reformulate to reduce levels of nutrients of concern [28,37,41,45,46,47].

It is clear that in order to improve populations’ diets a range of policies and interventions will be needed, across multiple sectors. Implementation of front-of-pack nutrition labelling has been recommended as one element of such a multi-component approach. Implementation of front-of-pack labelling has been recommended, for example, as part of a WHO package of best buys to reduce unhealthy diet (in relation to salt specifically) [48] and to tackle childhood obesity [49]. In the Eastern Mediterranean Region, implementation of front-of-pack nutrition labelling was proposed to Member States as part of a package of policy priorities for preventing obesity and diabetes in 2017 [50].

In the Eastern Mediterranean Region, implementation of front-of-pack nutrition labelling has been extremely limited to date. Globally, many different approaches to front-of-pack labelling have been adopted and there is an opportunity for countries in the Region to learn from other countries’ experience. There are currently no international standards for front-of-pack nutrition labelling, but WHO has published draft guiding principles and a framework manual for front-of-pack labelling [20]. In addition, the Codex Committee on Food Labelling is now working to produce guidance on front-of-pack labelling. Neither document is likely to establish one single recommended front-of-pack labelling scheme.

### 3.2. Global Implementation of Front-of-Pack Nutrition Labelling

Globally, a 2019 review concluded that 32 governments had endorsed some form of front-of-pack label [38]. Although some schemes have been in place for many years, implementation has intensified in the last decade and particularly over the last five years for labels which include assessments of harmful levels of certain nutrients.

#### Types of Front-of-Pack Labelling Systems

Jones et al. identified 31 unique front-of-pack nutrition labels [38] and, according to a 2017 stocktake of front-of-pack labelling schemes completed for the electronic working group of the Codex Committee on Food Labelling, there is no single or predominant scheme [35].

There is some variation in the different schemes that exist. Some key criteria have been identified to help categorise the different type of schemes, and these are shown in Table 1.

There are several other characteristics of front-of-pack nutrition label systems that may impact on their effectiveness. These include visual aspects of the label design (e.g., size, positioning, legibility, visibility, use of colours, and wording) which ensure it is understandable to all population subgroups, the robustness of the nutrient profile model that underlies the labelling system, the regulatory framework, and the transparency and governance of the system’s development and implementation.

A 2019 review found that 26 of the 31 identified front-of-pack labels used interpretive elements. The majority of labels identified were implemented voluntarily, but 10 were implemented on a mandatory basis [38]. This is up from four mandatory schemes identified by Codex in 2017 [35].

### 3.3. Implementation of Front-of-Pack Nutrition Labelling the WHO Eastern Mediterranean Region

Implemented front-of-pack nutrition labelling schemes were identified in three countries in the Region and three further schemes are under development.

The schemes identified in the Region fall into three categories:

- The nutrient-specific traffic light labelling (Islamic Republic of Iran, Kingdom of Saudi Arabia, United Arab Emirates);
- The Nutri-Score summary graded label (Morocco);
- Health or endorsement logos (Abu Dhabi and Tunisia).

Table 2 summarizes the three different types of scheme.

#### 3.3.1. Traffic Light Labelling Schemes

Three countries in the Region have introduced, or are in the process of introducing, traffic light labelling schemes, which use the red, amber, green colouring of road traffic lights to highlight low, medium, and high levels of nutrients. In general, research has found that traffic light labelling enhances consumer understanding of nutritional quality of foods compared to back-of-pack labelling and some other forms of front-of-pack nutrition label [15,17,39,40,51,52].

Iran introduced traffic light nutrition labelling for front of packs in 2014. The labelling system—which covers energy, sugars, total fat, trans fats, and salt—was initially voluntary and has been mandatory since 2016. Between September 2015 and September 2016, it was reported that 73% of locally produced and 61% of imported packaged foods sold in retail chains in Tehran carried the traffic light labels [53]. By May 2017 it was reported that 80% of foods were labelled with traffic lights [54]. A better-for-you award for healthier foods within food categories—the green apple—is also in use.

Saudi Arabia introduced traffic light labelling in 2018. The system is adapted from the UK traffic light system, and uses the UK’s thresholds for fat, saturated fat, total sugars, and salt but only on a 100 g/mL basis. The Saudi Food and Drug Authority initiated the measure as part of its efforts to promote public health and enable consumers to make healthier food choices, in order to prevent obesity and deal with the burden of NCDs across the country. In addition, as the traffic light label is being introduced for front-of-pack, the back-of-pack nutrient declaration is being strengthened, with a mandatory requirement to declare added sugars. The system is initially being introduced as a voluntary approach but may become mandatory depending on public demand and food industry uptake of the voluntary labelling.

The United Arab Emirates has announced introduction of front-of-pack traffic light labelling for fat, saturated fat, sugars, and salt levels on prepackaged foods as part of its National Program for Happiness and Wellbeing. The labelling scheme has been developed through cooperation between the Nutrition Department of the Ministry of Health and the Standards and Metrology Organization. Implementation will be initially implemented on a voluntary basis from 2020 but will become mandatory from January 2022 [55].

The systems adopted in the Region differ from the systems in use in Europe in several ways, the inclusion of trans fatty acids, for example, is one which is highly pertinent for the Eastern Mediterranean Region.

#### 3.3.2. Nutri-Score Summary Logo

The five-colour summary score label ranks foods from A (green) to E (red) according to an overall score. Letters are included because of concerns about accessibility for people who are colour blind and to allow for a grey-scale basis in the exceptional case of black and white packages.

The score is based on quantities of nutrients to limit (calories, sugars, saturated fat, and sodium) and foods and nutrients to encourage (fruits, vegetables, pulses, nuts, fibre, and protein) in a product, based on the composition for 100 g/mL. A high score on positive elements (nutrients to encourage) cannot negate a high score for negative elements.

The Nutri-Score interpretive label has been adopted, on a voluntary basis, by governments in France, Belgium, Spain, and Germany. Although Nutri-Score is a relatively young labelling system, in research to date it has consistently performed better than all other front-of-pack nutrition labels in facilitating consumer understanding of the nutritional quality of foods [52,56,57] and in influencing purchases towards healthier choices according to Nutri-Score criteria [58].

A study is underway in Morocco to evaluate the perception and the objective understanding of five different front-of-pack nutrition logos, including Nutri-Score, by Moroccan consumers. The Moroccan Nutrition Program and action plan 2017–2021 for reducing consumption of salt, sugar, and fat announced that a label or logo to describe the overall nutritional quality of foods would be introduced by developing legislation.

For the development of the Nutri-Score model, there has been extensive research and consumer testing. It is not necessary to repeat all of this developmental research, but further consumer testing in the specific context of the Region would be useful. Such testing of consumer response to front-of-pack labels is being conducted in Morocco and existing tools and study protocols are available for adaptation in the Region.

As with other front-of-pack labelling systems, effectiveness of Nutri-Score would be enhanced by implementation on a mandatory basis across all prepackaged foods.

#### 3.3.3. Health (Endorsement) Logos

Front-of-pack labels that provide an at-a-glance indication of healthier foods within categories are generally referred to as health logos or endorsement logos. There are examples of health logos in use, or under development, within the Eastern Mediterranean Region.

Health logos only apply to foods that meet predefined nutrient criteria. They provide an indication of healthier foods within categories, but do not feature less healthy foods and do not usually enable consumers to make comparisons between food categories. For this reason, they are sometimes considered to be health claims, rather than front-of-pack labelling [20,22].

Where this is the only scheme in place, consumers have no supplementary information about the majority of foods on the market, including those high in fat, sugar, and/or salt. Indeed, criteria used to define healthier foods are usually quite strict, therefore applying only to a minority of foods. Research suggests that consumers respond more to labels that communicate about less healthy aspects of foods (negative in tone) than positive aspects [59]. Consumer awareness of well-established logos can be very high (there is over 80% recognition of the KeyHole in Nordic countries), although research suggests that understanding of health logos can be poor [18,34], and that people may think foods carrying the logo are healthier than they actually are. Countries have attempted to tackle this in a number of ways (e.g., restricting the foods on which the logo can appear, defining stricter nutrient criteria) [34].

Research suggests that implementation of a health logo front-of-pack scheme is likely to have less impact than, for example, traffic lights or Nutri-Score [60]. However, for countries that wish to introduce this form of labelling, attention to a number of important considerations would maximise effectiveness. It is important that such logos are underpinned by robust nutrient criteria and that they have a visual format that is meaningful and recognisable for consumers in the local context.

In Abu Dhabi, a health logo scheme, called Weqaya, has been in place on a voluntary basis since 2015 [61]. Under this scheme, food businesses which meet particular food safety and hygiene requirements can apply to display the Weqaya (which means prevention in Arabic) logo on foods/dishes or meals that meet certain nutritional or compositional criteria for meals/takeaway food, children’s meals, or takeaway food and individual food items. These criteria cover issues such as cooking method (should not be deep fried), additives, portion size, marketing to children, and meal composition. Depending on the category, nutritional criteria are set for energy, total fat, saturated fat, trans fats, added sugar, salt/sodium, fruit/legumes/vegetables, fibre, and whole grains. For individual food products, there are 17 separate categories, with their own criteria. Furthermore, grocery stores and supermarkets have to meet specific marketing and health promotion requirements (provision of tasting of healthy items, healthy recipes, cooking/shopping tips, nutrition education materials; organization of events; store layout to encourage healthy purchases, and reduce unhealthy checkout purchases) if they want to promote the Weqaya label on foods.

In Tunisia, a front-of-pack health logo has been developed to help consumers to identify healthier food choices. The health logo label, which is in the form of a tick, includes the wording “National Strategy of Prevention and Control of Obesity”, referring to the overarching national nutrition program, because there is a high level of awareness of and confidence in this strategy. The tick format was selected after testing different formats with consumers, both adults and children, and finding that the tick had the highest level of acceptance. To carry the logo, foods need to meet nutrient profile criteria derived from three sources: The WHO Regional Nutrient Profile model [62]; the SAIN, LIM model [63,64]; and WHO guidelines. Foods that meet the criteria of all three approaches can qualify for the Tick logo. A mass media campaign is planned to promote awareness and use of the label, once the revised decree is published and roll out will begin on a very limited number of foods.

## 4. Discussion

Implementation of front-of-pack labelling in the Eastern Mediterranean Region remains limited, but three types of scheme were identified as having been implemented or at an advanced stage of development. The advantages and disadvantages of the three types of scheme—traffic lights; Nutri-Score; health logos—are summarized in Table 3. It is important to note that this discussion is limited to the types of scheme under development in the Eastern Mediterranean Region and there are many other types of front-of-pack nutrition labels in use, globally. Warning labels on foods which are high in energy, saturated fat, sugars, or sodium, for example, were introduced in Chile in 2016, and have since been adopted in Peru and Uruguay. In addition, the interpretive Health Star Rating system in use in Australia and New Zealand is not discussed in detail in this paper.

There is a growing evidence base in support of front-of-pack nutrition labelling and an increasing number of country examples of effective implementation. With all schemes, the effectiveness depends on exactly how it is implemented, including the governance and transparency of the development and implementation, and, to a large degree, on the validity of the nutrient profile model which underpins the labelling scheme.

Gaps remain in the evidence base, however, and as yet there remains little research in real world settings and there is no definitive evidence on which specific scheme is most effective. The most appropriate front-of-pack labelling scheme may vary from country to country, therefore, and policy makers need to choose the scheme most suitable to the particular national context.

The current proliferation of schemes and mixed evidence base can be confusing for policymakers and for this reason WHO is developing global guiding principles for front-of-pack labelling and Codex is discussing front-of-pack labelling and is expected to produce guidance.

Examination of the existing evidence base and country experience with implementation of front-of-pack labelling may provide some pointers for consideration by policymakers seeking to take action in this area in the Eastern Mediterranean Region.

Consumers appear to prefer interpretive labels to informative (or reductive) labelling [34,65]. Research suggests that labels which help consumers interpret nutrition information are more likely to have an impact on consumer understanding and food choices than informative labels [13,15,16,17,23]. Continued exposure to interpretive labels also has the potential to improve people’s literacy about the nutritional quality of foods [66] and interpretive labels may be able to reach groups with poor nutritional knowledge and unhealthy diets [42,44].

Research also suggests that consumers are most concerned to have information about which foods to consume less of or which foods have high levels of nutrients for which consumption should be limited [14,34,59]. Labels that include indications of the presence of high levels of fat, sugar or salt, or which give a graded overall score, are more likely to be effective than labels which only highlight healthier foods (positive judgement only) [34,65].

Front-of-pack labels should be designed to enable at-a-glance decisions about potential purchases. It is important that front-of-pack labels simplify the complex information provided in nutrient declarations, which is known to be difficult to comprehend [13,16,17,18]. Previous research tended to suggest that nutrient-specific schemes were better, but emerging evidence suggests that summary systems are easy to understand across all groups [39,42,45,51].

Inclusion of an aid to interpretation such as colour-coding, graphics, or interpretive text is likely to improve comprehension of the labels [13,15,24]. Green and red colours have been shown to be a key aspect to enhancing understanding [24,67,68] and the incorporation of red appears to have a more powerful influence than appearance of green [59]. There are concerns, however, that the information must be accessible to people who suffer from colour-blindness, which means that dependence on colour alone can be problematic.

Experience also demonstrates the importance of front-of-pack labelling schemes being underpinned by robust nutrient profile models, appropriate to the national context. Nutrient criteria need to reflect up-to-date guidelines if any changes in consumer behaviour are to result in healthier diets and be translated into health benefits, particularly reducing the risk of diet-related NCDs. The criteria underpinning the scheme also need to be transparent—a lack of transparency about the nutrient criteria can undermine confidence in the schemes.

Unless there are standardised serving sizes, there is the potential for consumers to be misled by figures expressed on a per serving basis if declared serving sizes set by manufacturers are smaller than actual portions consumed and vary across brands. This suggests that nutrient criteria should be based on nutrients per 100 g, rather than per serving, where countries do not have standard serving sizes.

Governments can choose to require companies to adopt front-of-pack labelling through legislation, or they may decide to define a scheme but leave implementation optional (or only mandatory in certain conditions or for some foods). Experience suggests that voluntary schemes do not always achieve sufficient coverage of the products on the market [34,66]. Five years after adoption of the government-led voluntary Health Star Rating in Australia the logo featured on 31% of products [69], while after two years in New Zealand only 5.3% of the products on retail shelves were labelled with the logo [45]. According to official estimates, by September 2019, two years after adoption, companies voluntarily applying the Nutri-Score label in France covered 25% of processed food products on the market [70]. Even in countries where a voluntary scheme has higher penetration, there can be distortion because less healthy products are less likely to carry front-of-pack labels. In the United Kingdom, for example, the Department of Health estimates that the voluntary scheme has been adopted by two-thirds of the packaged food and drink market [43], and historically penetration has been lower in categories where there are more foods high in fat, sugar or salt (meat products, pastry products, pizza, and prepared meals) [71]. Similarly, in Australia more than three quarters (76.4%) of foods displaying a Health Star Rating label had three or more stars [72]. Where voluntary approaches are pursued, there are approaches that can be used to maximize effectiveness and/or ensure widespread uptake and penetration of the scheme (e.g., defining only one single type of front-of-pack label to be permitted; setting a condition that any use of the front-of-pack label requires manufacturers to use the label across their brand and product range).

Irrespective of whether a scheme is mandatory or voluntary, the process to develop it should be transparent and government-led to ensure independence and maximize credibility. It is important that research and testing findings are published and that the scope, aims, and objectives of front-of-pack labelling are transparent. The process clearly requires engagement with industry, consumers and other stakeholders, but governmental processes should incorporate robust safeguards from conflicts of interest.

It is important for front-of-pack nutrition labelling to be implemented as part of a comprehensive strategy to promote healthy diets and prevent obesity and diet-related NCDs. In the Eastern Mediterranean Region, the countries that have introduced, or are in the process of introducing, front-of-pack labels are doing so as part of a broader approach to promoting healthy diets. These programmes also include, variously, nutrition labelling on menus, taxation of sugar-sweetened beverages, regulatory limits on trans fatty acids, nutrition standards for food in schools, and/or hospitals, government-led reformulation programmes and behaviour change communication. The effectiveness of front-of-pack labelling is likely to be enhanced by nutrition education, while front-of-pack labels can in themselves be educational and may help people to translate learning into action. Consumer understanding of front-of-pack labels is likely to increase over time and will be enhanced if there is one single front-of-pack labelling scheme to minimize confusion.

As countries prepare to introduce a front-of-pack labelling scheme, there are a number of steps that can help to pre-empt any trade complaints [73]. It is important for policy makers to engage with the legal sector to ensure that policy objectives are framed in a strategic way—as part of a comprehensive response to a public health problem—and that the justification for why the measure is necessary, effective, and proportional is clearly set out. Inclusion of explicit references to WHO guidelines, guiding principles, and recommendations is advisable and it may be useful to cite the precautionary principle.

## 5. Conclusions

In conclusion, policies to promote the implementation of simplified nutrition information on the front of food packages can be an important element of strategies which aim to improve population diets. Implementation in the Eastern Mediterranean Region has been very limited to date, although a small number of countries are now leading the way.

There are currently three types of front-of-pack labels in use or under development in the Eastern Mediterranean Region. Namely, traffic lights systems, Nutri-Score, and health logos. This paper has summarized some of the advantages and disadvantages of these systems and—through a review of existing research and evidence from country implementation—summarized some emerging messages on implementation for any countries deciding to implement one of these schemes in the Region.

Access to simplified nutrition information is particularly important in this Region, where many countries rely heavily on imported food. There is considerable scope for countries to work together and to exchange experiences, share tools and methods, and collaborate in other ways on the implementation of front-of-pack labelling. Establishment of a regional network on front-of-pack nutrition labelling could be a helpful step to facilitate such cooperation across the Region.

## Figures and Tables

**Table 1:** Key characteristics for categorizing front-of-pack labelling schemes.

Characteristics	Options	Illustrative Examples
Interpretive or informative provision of information	Interpretive schemes provide information to help consumers understand how healthy/unhealthy a food product is. This is often conveyed through use of colour coding, graphic symbols, or interpretive words (such as ‘high in’ or ‘low in’).	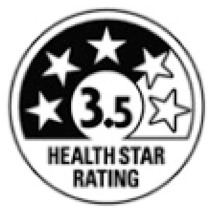 Health Star Rating system (Australia/New Zealand)
	Informative schemes (sometimes known as reductive) provide factual information, with no specific judgement or guidance about the nutritional quality of a food product.	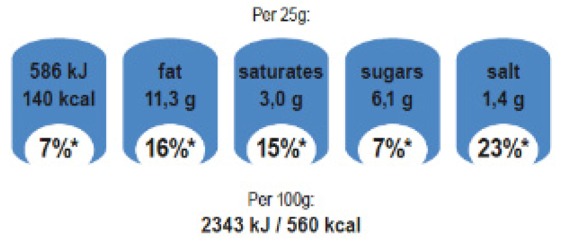 EU food industry Reference Intakes
	Hybrid schemes provide a mix of factual information and interpretive elements.	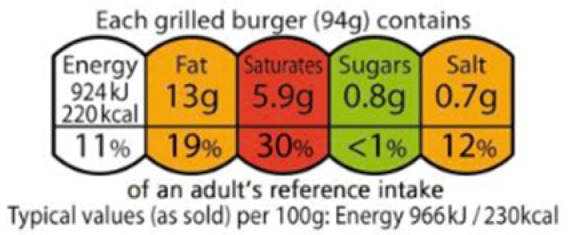 UK traffic light labels combine informative reference intakes and interpretive colour coding
Summary or nutrient based	Summary schemes show an overall indicator of the healthiness of a product, based on a combination of several nutritional criteria.	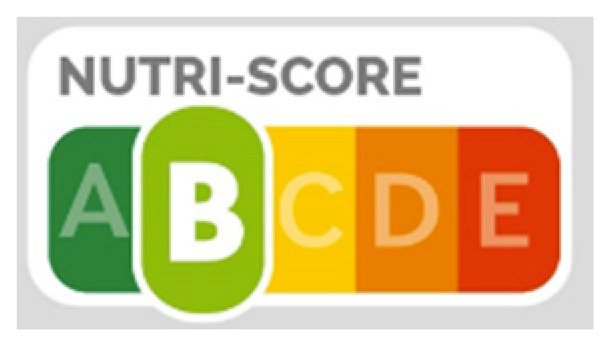 Nutri-Score (France, Belgium, Spain, Germany)
	Nutrient-specific schemes provide information on a set of nutrients.	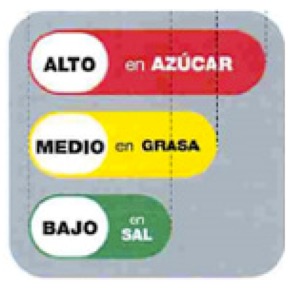 Traffic light labels (Ecuador)
Tone of judgement (for interpretive schemes)	Labels that only identify products of a higher nutritional quality (i.e., positive judgement only). These are often referred to as ‘endorsement logos’ and are sometimes considered to be health claims rather than nutrition labels [22].	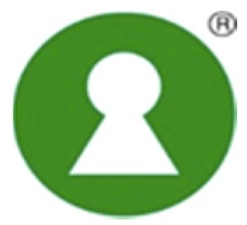 Nordic Keyhole (Sweden, Denmark, Norway, Lithuania)
	Labels that provide a graded indicator of nutritional quality or indicate levels of both nutrients/ingredients that are considered healthier and those for which consumption should be limited (positive and negative).	See Nutri-Score and the Health Star Rating
	Labels that only identify foods which have high levels of less healthy nutrients/ingredients for which consumption should be limited (negative only).	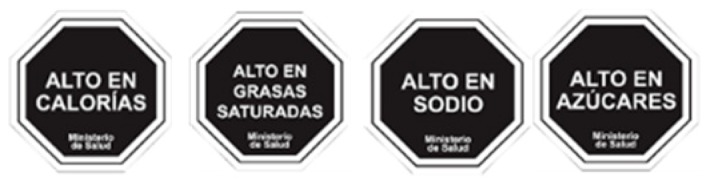 Warning labels (Chile)
Mandatory or voluntary implementation	Mandatory schemes require companies to include the specified labels on food packs. Some schemes apply to all foods, others to specific categories.	Mandatory schemes are in place in, for example, in Chile, Ecuador, Finland, Mexico, Sri Lanka, Thailand, and Uruguay.
	Under voluntary schemes companies can choose whether or not to use the labels. In some cases, government specifies the type of label to be used, although their use is optional. Other voluntary schemes may be driven by industry or other stakeholders, and manufacturers can choose whether or not to use them.	Governments have endorsed voluntary schemes in, for example, Australia, Croatia, Finland, France, Iceland, New Zealand, Norway, Singapore, South Korea, Sweden, and United Kingdom.
Range of nutrients and ingredients included	Schemes vary from those that focus on a nutrient/ingredient alone (e.g., energy or salt) to those which cover a wide range of nutrients/ingredients. Most commonly included components are sodium/salt, energy, total sugars, saturated fat, total fat, trans fatty acids, and added sugars.	Nutri-Score, for example, is based on calories, sugars, saturated fat and sodium, as well as presence of fruits, vegetables, pulses, nuts, fibre, and protein UK traffic light labels cover energy, saturated fat, sugars, and salt
Reference amount for nutrients	Nutrient calculations and/or declarations can be based on: Per 100 g/100 mL Per serving/portion size	The Reference Intakes label above, used in the European Union, is based on per serving. Nutri-Score, the Health Star Rating, and Chile’s warning labels, for example, are based on 100 g/100 mL. Some systems, e.g., UK traffic lights, use a combination of ‘per serving’ and ‘per 100 g/100 mL’ information.

**Table 2:** Different types of front-of-pack nutrition labelling schemes in use or under development in the WHO Eastern Mediterranean Region.

Nature of Information Provided	Summary Indicator/Nutrient Specific	Tone	Examples	Comments
Interpretive (providing information as guidance to help consumers understand the relative healthiness of food products)	Summary indicator	Positive	Tunisia: 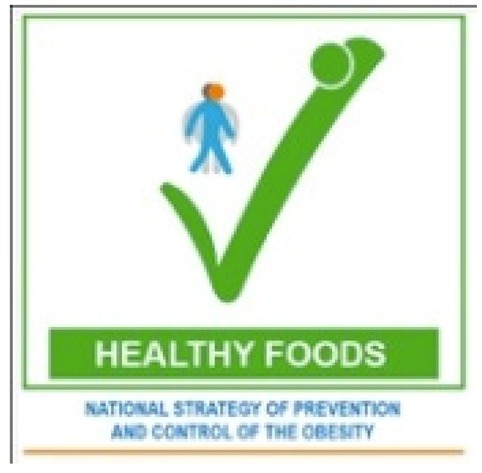 Abu Dhabi: 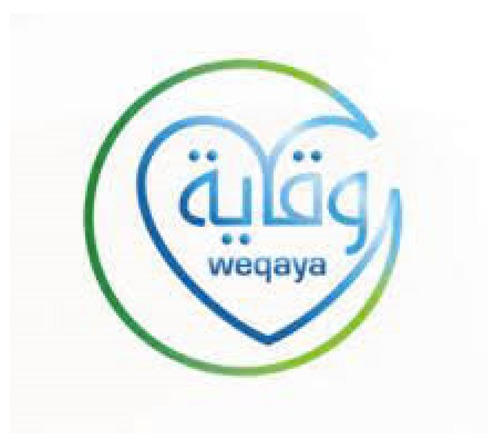	Logos which are positive summary indicators indicate healthier foods within categories. These are often referred to as endorsement or health logos (and these are often defined as ‘health claims’ rather than front-of-pack labelling). Tunisia is introducing a health logo label in the form of a tick. A health logo scheme, called Weqaya, has been implemented on a voluntary basis in Abu Dhabi since 2015.
		Positive and negative	Under development in Morocco: 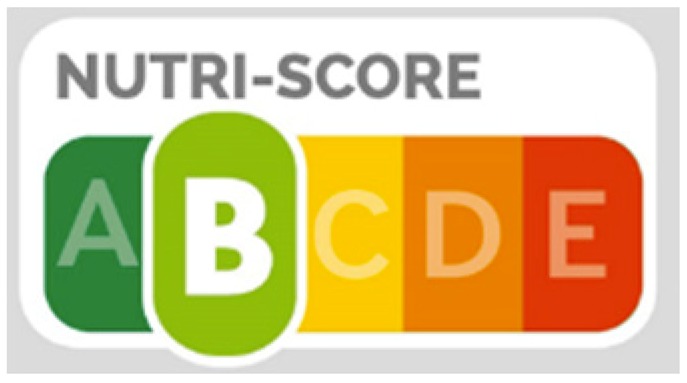	Summary indicators which cover both positive and negative elements give an overall rating of how healthy a food is. Morocco is conducting research studies to explore implementation of one summary indicator, Nutri-Score, under the Moroccan national 2017–2021 action plan for reducing consumption of salt, sugar and fat, and the national programme of nutrition.
	Nutrient specific	Positive	In general, these are health claims, rather than nutrition labels.	
		Positive and negative	Islamic Republic of Iran: 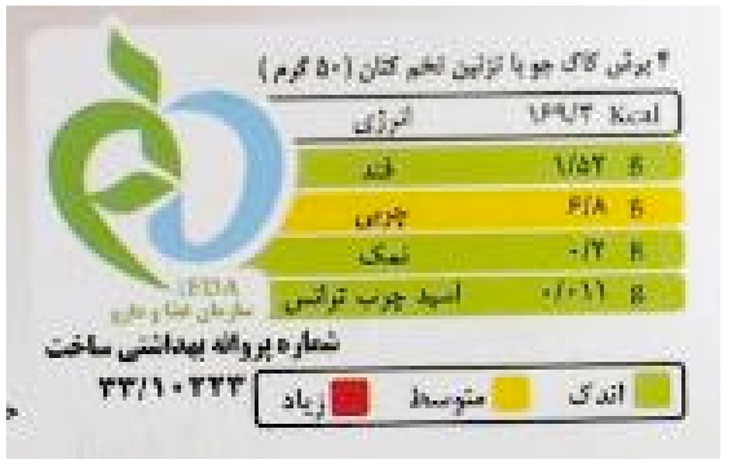 Kingdom of Saudi Arabia: 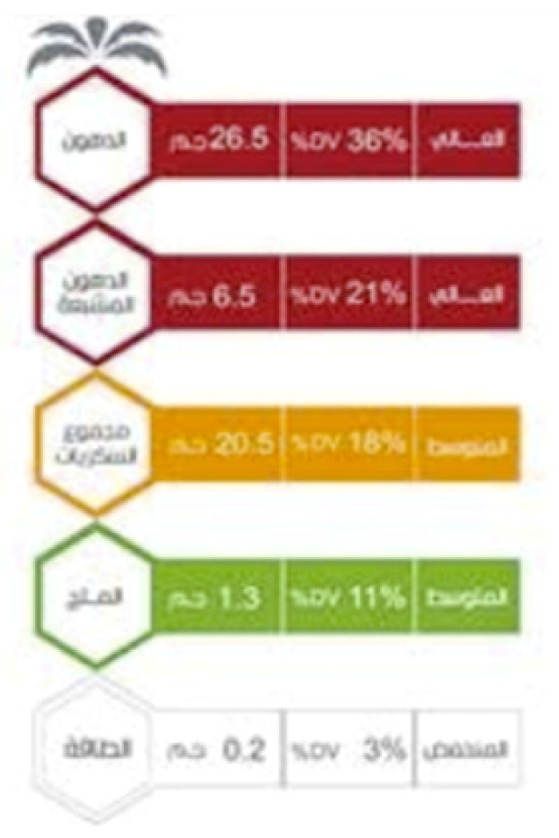	These schemes apply traffic light colour coding (red, amber, green) to several nutrients to indicate the relative healthiness of those nutrient levels. Iran introduced voluntary traffic light labels in 2014, and these have been mandatory since 2016. The Saudi Food and Drug Authority introduced traffic light labelling in 2018, initially on a voluntary basis. Implementation of traffic lights from 2020 has also been announced in the United Arab Emirates, initially on a voluntary basis, but becoming mandatory by 2022.
		Negative	Nutrient specific negative labels are “warning labels”, as implemented in, for example, Chile.	No implementation of warning labels identified in the Eastern Mediterranean Region.
Informative (provide factual information, with no guidance on interpretation)	Nutrient specific		These include labels which show the percentage of guideline daily amounts of particular nutrients provided by the food. They do not include any colour-coding or wording to help consumers interpret the information.	No implementation of government-led schemes which only provide such information has been identified in the Eastern Mediterranean Region

**Table 3:** Strengths and weaknesses of three different types of front-of-pack labels currently being adopted in the Eastern Mediterranean.

Type of Front-of-Pack Label	Strengths	Weaknesses
Traffic light labelling	✓ Simplified information that is easy to understand ✓ Interpretive to aid healthy choices ✓ Includes an indication of healthy foods and less healthy foods ✓ Colour-coding aids understanding, inclusion of ‘reds’ is particularly useful ✓ Design based on an already understood concept (traffic lights) ✓ Enables comparisons between food categories, within categories and within a specific food type ✓ Allows people to pay attention to particular nutrients of concern/interest ✓ Examples of country implementation and well-established ✓ Potential to drive reformulation of both healthier and less healthy products	✕ Not as simple as overall summary system scores (e.g., Nutri-Score) ✕ Consumers may have difficulty identifying the healthiest option when there is trade-off between nutrients ✕ Focuses only on the “negative” nutrients/components ✕ Inclusion in some systems of the noninterpretive element (e.g., percentage of reference intakes) could be confusing ✕ Unless implementation is mandatory, it is more likely to be used on healthier products
Nutri-Score	✓ Simplified information that is easy to understand ✓ Interpretive to aid healthy choices ✓ Includes an indication of healthy foods and less healthy foods ✓ Provides a single overall score for a food; does not require any understanding of nutrients ✓ Design based on an already understood concept in Europe (appliance energy ratings) ✓ The nutrient profile takes into account both “negative” and “positive” components of a food ✓ Enables comparisons between food categories, within categories and within a specific food type ✓ Strong evidence base from extensive research and testing during development ✓ Research suggests it is understood by all population groups, including those who normally do not read labels or who have poor diets ✓ Potential to drive reformulation of both healthier and less healthy products	✕ Does not allow people to pay attention to particular nutrients of concern/interest ✕ A relatively new labelling system which has relatively limited country implementation experience ✕ Unless implementation is mandatory, it is more likely to be used on healthier products
Health or endorsement logos	✓ Simplified information that is easy to understand ✓ Interpretive to aid healthy choices ✓ Provides a simple logo and does not require any understanding of nutrients ✓ Designs are often based on readily understood visual concepts (e.g., tick, heart) ✓ Enables comparisons within categories and within a specific food type ✓ May meet less resistance than a labelling system which includes “negative” as well as “positive” evaluation of foods ✓ Potential to drive reformulation of healthier products	✕ Does not include an indication of less healthy foods ✕ Does not cover most foods on the market, including those high in fat, sugar or salt ✕ Does not always enable comparisons between foods categories ✕ Does not allow people to pay attention to particular nutrients ✕ Consumers may overestimate the healthiness of products carrying the logo ✕ Less likely to drive reformulation of less healthy products

## References

[1] World Health Organization (2014). Global Status Report on Noncommunicable Diseases 2014.

[2] Institute for Health Metrics and Evaluation GBD Compare Data Visualization. https://vizhub.healthdata.org/gbd-compare/.

[3] World Health Organization (2004). Global Strategy on Diet, Physical Activity and Health.

[4] World Health Organization (2014). Comprehensive Implementation Plan on Maternal Infant and Young Child Nutrition.

[5] Food and Agriculture Organization of the United Nations, World Health Organization (2014). Second International Conference on Nutrition: Framework for Action.

[6] World Health Organization (2013). Global Action Plan for the Prevention and Control of Noncommunicable Diseases 2013–2020.

[7] WHO Regional Office for the Eastern Mediterranean (2018). Regional Framework for Action on Obesity Prevention 2019–2023.

[8] Codex Alimentarius Commission (2017). Guidelines on Nutrition Labelling (CAC/GL-1985).

[9] World Health Organization (2018). Global Nutrition Policy Review 2016–2017.

[10] Musaiger A., Al-Hazzaa H., Takruri H., Mokhatar N. (2012). Change in nutrition and lifestyle in the Eastern Mediterranean Region: Health Impact. J. Nutr. Metab..

[11] WHO Regional Office for the Eastern Mediterranean Region (2011). Regional Strategy on Nutrition 2010–2019 and Plan of Action.

[12] World Health Organization (2009). Regional Strategy on Nutrition 2010–2019.

[13] Campos S., Doxey J., Hammond D. (2011). Nutrition labels on pre-packaged foods: A systematic review. Public Health Nutr..

[14] Drichoutis A.C., Lazaridis P., Nagya R. (2006). Consumers’ use of nutritional labels: A review of research studies and issues. Acad. Mark. Sci. Rev..

[15] Volkova E., Ni Mhurchu C. (2015). The influence of nutrition labeling and point-of-purchase information on food behaviours. Curr. Obes. Rep..

[16] Cowburn G., Stockley L. (2005). Consumer understanding and use of nutrition labelling: A systematic review. Public Health Nutr..

[17] Hawley K.L., Roberto C.A., Bragg M.A., Liu P.J., Schwartz M.B., Brownell K.D. (2012). The science on front-of-package food labels. Public Health Nutr..

[18] Ni Mhurchu C., Gorton D. (2007). Nutrition labels and claims in New Zealand and Australia: A review of use and understanding. Aust. N. Z. J. Public Health.

[19] Codex Committee on Food Labelling (2017). Comments from International Association of Consumer Food Organisations on Proposal for New Work Concerning a Global Standard for Front of Pack Interpretive Nutrition Labelling.

[20] World Health Organization (2019). Draft WHO Guiding Principles and Framework Manual for Front-of-Pack Labelling for Promoting Healthy Diets.

[21] Talati Z., Egnell M., Hercberg S., Julia C., Pettigrew S. (2019). Consumers’ perception of five front-of-package nutrition labels: An experimental study across 12 countries. Nutrients.

[22] Rayner M., Wood A., Lawrence M., Mhurchu N.M., Albert J., Barquera S., Friel S., Hawkes C., Kelly B., Kumanyika S. (2013). Monitoring the health-related labelling of foods and non-alcoholic beverages in retail settings. Obes. Rev..

[23] Cecchini M., Warin L. (2016). Impact of food labelling systems on food choices and eating behaviours: A systematic review and meta-analysis of randomized studies. Obes. Rev..

[24] Hersey J., Wohlgenant K., Arsenault J., Kosa K., Muth M. (2013). Effects of front-of-package and shelf nutrition labeling systems on consumers. Nutr. Rev..

[25] Storcksdieck genannt Bonsmann S., Wills J. (2012). Nutrition Labeling to Prevent Obesity: Reviewing the Evidence from Europe. Curr. Obes. Rep..

[26] Crockett R., King S., Marteau T., Prevost A., Bignardi G., Roberts N., Stubbs B., Hollands G., Jebb S. (2018). Nutritional labelling for healthier food or non-alcoholic drink purchasing and consumption. Cochrane Database Syst. Rev..

[27] Grunert K., Wills J., Fernández-Celemín L. (2010). Nutrition knowledge, and use and understanding of nutrition information on food labels among consumers in the UK. Appetite.

[28] Vyth E. (2010). Front-of-pack nutrition label stimulates healthier product development: A quantitative analysis. Int. J. Behav. Nutr. Phys. Act..

[29] van Kleef E., Dagevos H. (2015). The growing role of front-of-pack nutrition profile labeling: A consumer perspective on key issues and controversies. Crit. Rev. Food Sci. Nutr..

[30] Talati Z., Pettigrew S., Neal B., Dixon H., Hughes C., Kelly B. (2017). Consumers’ responses to health claims in the context of other on-pack nutrition information: A systematic review. Nutr. Rev..

[31] Temple N.J., Fraser J. (2014). Food labels: A critical assessment. Nutrition.

[32] Graham D.J., Orquin J.L., Visschers V.H.M. (2012). Eye tracking and nutrition label use: A review of the literature and recommendations for label enhancement. Food Policy.

[33] Hieke S., Taylor C.R. (2012). A critical review of the literature on nutrition labelling. J. Consum. Aff..

[34] Kelly B., Jewell J. (2018). What Is the Evidence on the Policy Specifications, Development Processes and Effectiveness of Existing Front-of-Pack Food Labelling Policies in the Who European Region? Health Evidence Network (Hen) Synthesis Report 61.

[35] Codex Committee on Food Labelling Electronic Working Group (2017). Discussion Paper on Consideration of Issues Regarding Front-of-Pack Nutrition Labelling.

[36] World Cancer Research Fund International (2019). Building Momentum: Lessons on Implementing a Robust Front-of-Pack Food Label.

[37] Shangguan S., Afshin A., Shulkin M., Ma W., Marsden D., Smith J., Saheb-Kashaf M., Shi P., Micha R., Imamura F. (2019). A meta-analysis of food labeling effects on consumer diet behaviors and industry practices. Am. J. Prev. Med..

[38] Jones A., Neal B., Reeve B., Mhurchu C.N., Thow A.M. (2019). Front-of-pack nutrition labelling to promote healthier diets: Current practice and opportunities to strengthen regulation worldwide. BMJ Glob. Health.

[39] Neal B., Crino M., Dunford E., Gao A., Greenland R., Li N., Ngai J., Ni Mhurchu C., Pettigrew S., Sacks G. (2017). Effects of different types of front-of-pack information on the healthiness of food purchases–a randomized controlled trial. Nutrients.

[40] Jones G., Richardson M. (2007). An objective examination of consumer perception of nutrition information based on healthiness ratings and eye movements. Public Health Nutr..

[41] Friere W., Water W., Rivas-Mariño G., Nguyen T., Rivas P. (2016). A qualitative study of consumer perceptions and use of traffic light food labelling in Ecuador. Public Health Nutr..

[42] Julia C., Hercberg S. (2017). Development of a new front-of-pack nutrition label in France: The five-colour Nutri-Score. Public Health Panor..

[43] Skotarenko L. The UK’s Voluntary Front of Pack Nutrition Labelling Scheme. Proceedings of the European Commission Meeting.

[44] Leclerc E. (2018). Leclerc et Marque Repère Confirmed les Résultats Positifs du Nutriscore.

[45] Ni Mhurchu C., Eyles H., Choi Y. (2017). Effects of a voluntary front-of-pack nutrition labelling system on packaged food reformulation: The Health Star Rating in New Zealand. Nutrients.

[46] Young L., Swinburn B. (2002). Impact of the Pick the Tick food information programme on the salt content of food in New Zealand. Health Promot. Int..

[47] Eckel R., Borra S., Lichtenstein A., Yin-Piazza S. (2007). Understanding the complexity of trans fatty acid reduction in the American diet: American Heart Association Trans Fat Conference 2006: Report of the Trans Fat Conference Planning Group. Circulation.

[48] World Health Organization (2017). Tackling NCDS: ‘Best Buys’ and Other Recommended Interventions for the Prevention and Control of Noncommunicable Diseases.

[49] World Health Organization (2016). Report of the Commission on Ending Childhood Obesity.

[50] World Health Organization (2017). Proposed Policy Priorities for Preventing Obesity and Diabetes in the Eastern Mediterranean Region. EMRO Technical Publications Series 46.

[51] Ni Mhurchu C., Volkova E., Jiang Y., Eyles H., Michie J., Neal B., Blakely T., Swinburn B., Rayner M. (2017). Effects of interpretive nutrition labels on consumer food purchases: The Starlight randomized controlled trial. Am. J. Clin. Nutr..

[52] Egnell M., Talati Z., Hercberg S., Pettigrew S., Julia C. (2018). Objective understanding of front-of-package nutrition labels: An international comparative experimental study across 12 countries. Nutrients.

[53] Azizollaah Z., Dinarvand R., Hosseini H. (2017). Nutritional traffic light labeling and taxation on unhealthy food products in Iran: Health policies to prevent non-communicable diseases. Iran. Red Crescent Med. J..

[54] Tehran Times ‘Traffic light’ labels to guide Iranians toward healthier food. May 2017, 9:6. Tehran Times. https://www.tehrantimes.com/news/413178/Traffic-light-labels-to-guide-Iranians-toward-healthier-food.

[55] National Program for Happiness and Wellbeing, 2019. To promote Healthy Lifestyle and Wellbeing the UAE National Program for Happiness and Wellbeing launches Nutrition Labelling Policy. https://www.hw.gov.ae/en/news/to-promote-healthy-lifestyle-and-wellbeing-in-the-uae-national-program-for-happiness-and-wellbeing-launches-nutrition-labelling-policy.

[56] Julia C., Etilé F., Hercberg S. (2018). Front-of-pack Nutri-Score labelling in France: An evidence-based policy. Lancet Public Health.

[57] Julia C., Péneau S., Buscail C., Gonzalez R., Touvier M., Hercberg S., Kesse-Guyot E. (2017). Perception of different formats of front-of-pack nutrition labels according to sociodemographic, lifestyle and dietary factors in a French population: Crosssectional study among the NutriNet-Santé cohort participants. BMJ Open.

[58] Crosetto P., Lacroix A., Muller L., Ruffieux B. (2019). Nutritional and economic impact of five alternative front-of-pack nutritional labels: Experimental evidence. Eur. Rev. Agric. Econ..

[59] Scarborough P., Matthews A., Eyles H., Kaur A., Hodgkins C., Raats M., Rayner M. (2015). Reds are more important than greens: How UK supermarket shoppers use the different information on a traffic light nutrition label in a choice experiment. Int. J. Behav. Nutr. Phys. Act..

[60] Ducrot P., Julia C., Méjean C., Kesse-Guyot E., Touvier M., Fezeu L., Hercberg S., Péneau S. (2016). Impact of different front-of-pack nutrition labels on consumer purchasing intentions: A randomized controlled trial. Am. J. Prev. Med..

[61] Abu Dhabi Quality and Conformity Council (2015). Specification for Using Weqaya Food Program (ADS13/2018).

[62] World Health Organization Regional Office for the Eastern Mediterranean (2017). Nutrient Profile Model for the Marketing of Food and Non-Alcoholic Beverages to Children in the WHO Eastern Mediterranean Region.

[63] AFSSA (2008). Définition de Profils Nutritionnels Pour L’accès aux Allégations Nutritionnelles et de Santé: Propositions et Arguments.

[64] Darmon N., Vieux F., Maillot M., Volatier J.-L., Martin A. (2009). Nutrient profiles discriminate between foods according to their contribution to nutritionally adequate diets: A validation study using linear programming and the SAIN, LIM system. Am. J. Clin. Nutr..

[65] Talati Z., Pettigrew S., Kelly B., Ball K., Dixon H., Shilton T. (2016). Consumers’ response to front-of-pack labels that vary by interpretive content. Appetite.

[66] Kanter R., Vanderlee L., Vandevijvere S. (2018). Front-of-package nutrition labelling policy: Global progress and future directions. Public Health Nutr..

[67] Rohr M., Kamm F., Koenigstorfer J., Groeppel-Klein A., Wentura D. (2015). The Color Red Supports Avoidance Reactions to Unhealthy Food. Exp. Psychol..

[68] Cabrera M., Machin L., Arrúa A., Curutchet M., Giménez A., Ares G. (2017). Nutrition warnings as front-of-pack labels: Influence of design features on healthfulness perception and attentional capture. Public Health Nutr..

[69] MP Consulting Health Star Rating System Five Year Review Draft Report. February 2019. MP Consulting. http://www.healthstarrating.gov.au/internet/healthstarrating/publishing.nsf/Content/D1562AA78A574853CA2581BD00828751/$File/Health-Star-Rating-System-Five-Year-Review-Draft-Report.pdf.

[70] Oqali Déploiment du Nutri-Score: Analyse à Partir des Données Transmises à l’Oqali. https://www.oqali.fr/content/download/3635/34510/version/1/file/Oqali+2019_Deploiement_du_Nutri_Score_analyse_a_partir_des_donnees_transmises_a_l_Oqali.pdf.

[71] Van Camp D., Souza-Monteiro D.M., Hooker N.H. Stop Or Go? How Is The Uk Food Industry Responding To Front-Of-Pack Nutrition Labels?. Proceedings of the 115th Joint EAAE/AAEA Seminar.

[72] Jones A., Shahid M., Neal B. (2018). Uptake of Australia’s Health Star Rating System. Nutrients.

[73] Thow A.M., Jones A., Hawkes C., Ali I., Labonté R. (2017). Nutrition labelling is a trade policy issue: Lessons from an analysis of specific trade concerns at the World Trade Organization. Health Promot. Int..

